# Institutionalizing evidence-based STEM reform through faculty professional development and support structures

**DOI:** 10.1186/s40594-022-00353-z

**Published:** 2022-05-12

**Authors:** Sreyasi Biswas, Rocio Benabentos, Eric Brewe, Geoff Potvin, Julian Edward, Marcy Kravec, Laird Kramer

**Affiliations:** 1 Taylor Institute for Teaching and Learning, University of Calgary, Calgary, Canada; 2 STEM Transformation Institute, Florida International University, 11200 SW 8th St, Miami, FL 33199 USA; 3 Department of Physics, Drexel University, Philadelphia, PA 19104 USA; 4 Department of Physics, Florida International University, Miami, FL 33199 USA; 5 Department of Mathematics and Statistics, Florida International University, Miami, FL 33199 USA; 6 Department of Biological Sciences, Florida International University, Miami, FL 33199 USA; 7 Academic Affairs, Florida International University, Miami, FL 33199 USA

**Keywords:** Instructional change, Professional development, Post-secondary faculty

## Abstract

**Background:**

Even though student-centered instruction leads to positive student outcomes, direct instruction methods are still prevalent. Multiple barriers prevent faculty from further adopting evidence-based student-centered practices and holistic approaches to faculty support are necessary to promote faculty change. The Collaborative for Institutionalizing Scientific Learning (CISL) is an HHMI-funded program to reform undergraduate science and mathematics education at a large Hispanic-Serving public research university. The program has established a Faculty Scholar support model to impact the number of science and mathematics faculty using evidence-based practices in their classrooms. Through this program, Scholars are selected to undertake a transformation of a course of their choice and conduct an assessment of the impact of the reform on students—while receiving multiple supports including summer salary, undergraduate Learning Assistants, professional development, course assessment and education research support, and opportunities to develop manuscripts on their course transformations.

**Results:**

CISL has supported over 40 Faculty Scholars in the transformation of both introductory and upper division biology, chemistry, physics and mathematics courses. Faculty are motivated to transform a course due to factors related to their own experiences and beliefs, their students’ needs, the course structure, and/or departmental elements. Quantitative analysis of the impact of the project on student success show that, overall, students in CISL-supported courses have higher passing rates compared to students in traditional classrooms. Survey and interviews of Faculty Scholars identified that the most valuable elements of the program were the personnel support from undergraduate Learning Assistants during reform implementation and guidance from the program’s Assistant Director during design, implementation and evaluation.

**Conclusions:**

The CISL program provides an example of significant effort sustained over several years to systematically improve the quality and culture of undergraduate education in a large research-intensive Hispanic Serving Institution. The program has had an overall positive impact on the professional development of Faculty Scholars and led to an increase in the number of STEM courses implementing evidence-based teaching practices, thus, taking a step towards solidifying a culture of evidence-based instructional strategies in STEM departments.

**Supplementary Information:**

The online version contains supplementary material available at 10.1186/s40594-022-00353-z.

## Introduction

There is an urgent need to increase the number and diversity of well-prepared qualified STEM professionals (Bradforth et al., 2015; National Academies of Sciences, Engineering and Medicine, 2011; National Research Council, 2012; Olson & Riordan, 2012). Recent calls have specifically noted the need for faculty to promote a change from traditional lecture-based teaching toward a more iterative and evidence-based student-centered approach (AAAS Annual Report, 2011; Auerbach & Schussler, 2017; Kuh, 2001; Woodin et al., 2010). These calls are supported by the increasing number of studies that indicate overall gains in student outcomes in student-centered environments that implement active learning strategies, when compared to traditional lecture-based classrooms (Armbruster et al., 2009; Chasteen et al., 2015; Freeman et al., 2014; Matz et al., 2018; Olson & Riordan, 2012; Prince, 2004; Wright, 2011). Active learning strategies have been correlated with increase in student mastery of course content, student passing rates, and student retention in the major, particularly for students from historically minoritized backgrounds (Freeman et al., 2014; Seymour, 2002). These studies provide strong evidence that student-centered teaching practices lead to increased positive student outcomes. Importantly, active learning has also been shown to have a positive impact on reducing achievement gaps observed for STEM students from minoritized backgrounds and is an important tool for diversifying the STEM workforce and providing more equitable access to STEM education (Theobald et al., 2020). However, despite these compelling results, recent studies have shown that lecture is still the most common instructor behavior (Benabentos et al., 2020; Eagan, 2016; Macdonald et al., 2005; Stains et al., 2018; Zieffler et al., 2012). A large-scale study of classroom observations of courses across multiple STEM disciplines showed that only 18% of the instructor observations could be classified as “student-centered” using active learning strategies in a large portion of the class. On average, lecture still took place in 50% of these “student-centered” class observation intervals (Stains et al., 2018). These results highlight that despite the mounting evidence, student-centered teaching practices are still not being widely adopted.

There are multiple barriers that prevent faculty from changing their mode of instruction and adopting evidence-based student-centered teaching practices (Henderson & Dancy, 2007). These barriers include faculty beliefs in traditional conceptions about teaching and learning (Gautreau & Novemsky, 1997; Hativa & Goodyear, 2002; Redish, 2004; Van Heuvelen, 1991); faculty satisfaction with traditional instruction (Gautreau & Novemsky, 1997; Van Heuvelen, 1991); and faculty lack of awareness of alternatives to lecture-based practices (Seymour, 2002; Simmons, 2006). In recent years, a substantial body of work has identified a number of additional barriers that play a significant role in impeding faculty from adopting evidence-based teaching approaches in their classrooms. Factors such as insufficient training in evidence-based teaching practices (Ebert-May et al., 2011; Handelsman et al., 2004; Hativa, 1995; Miller et al., 2000; Rushin et al., 1997; Yarnall et al., 2007), time for course reform (Shadle et al., 2017), and financial incentives to reform their teaching practices (Brownell & Tanner, 2012; Gess-Newsome et al., 2003) are among the most commonly cited barriers for faculty change. Of these, providing training in evidence-based teaching practices has been the focus of most of the current efforts to promote faculty pedagogical change (Henderson et al., 2010, 2011). Several national-level initiatives such as the NSF-funded FIRST IV program and the NAS/HHMI Summer Institutes for Undergraduate Biology Education are examples of interventions providing training to faculty of all levels. In addition, professional development workshops geared towards filling this gap have been shown to be effective in increasing the use of evidence-based teaching approaches (Bathgate et al., 2019; Owens et al., 2018). Financial incentives, such as salary compensation or supporting Teaching Assistants, have also shown to be effective in bringing about faculty change towards using more student-centered approaches in classrooms (Gess-Newsome et al., 2003; Matz et al., 2018). It is important to note that institutional and/or departmental support and a shared institutional vision can also help mitigate some of these barriers. A recent study by Bathgate et al. (2019) shows that faculty who perceive increased availability of institutional resources report implementing more evidence-based practices, even when challenges are present. There is now stronger evidence that changing the culture of undergraduate education towards more student-centered learning involves not just convincing individual faculty to change the way they teach but also providing faculty with appropriate training and institutional support structures that reflect the value of effective instructional practices (DeHaan, 2005; Seymour, 2002). To promote large-scale adoption of active-learning strategies across institutions, a systems approach needs to be considered, focusing on both individual faculty members as change agents as well as the institutional contexts and structures faculty are part of (Amundsen & Wilson, 2012; Austin, 2011; Henderson et al., 2010; Kezar, 2018; Porter et al., 2006; Seymour, 2002).

**Fig. 1:**
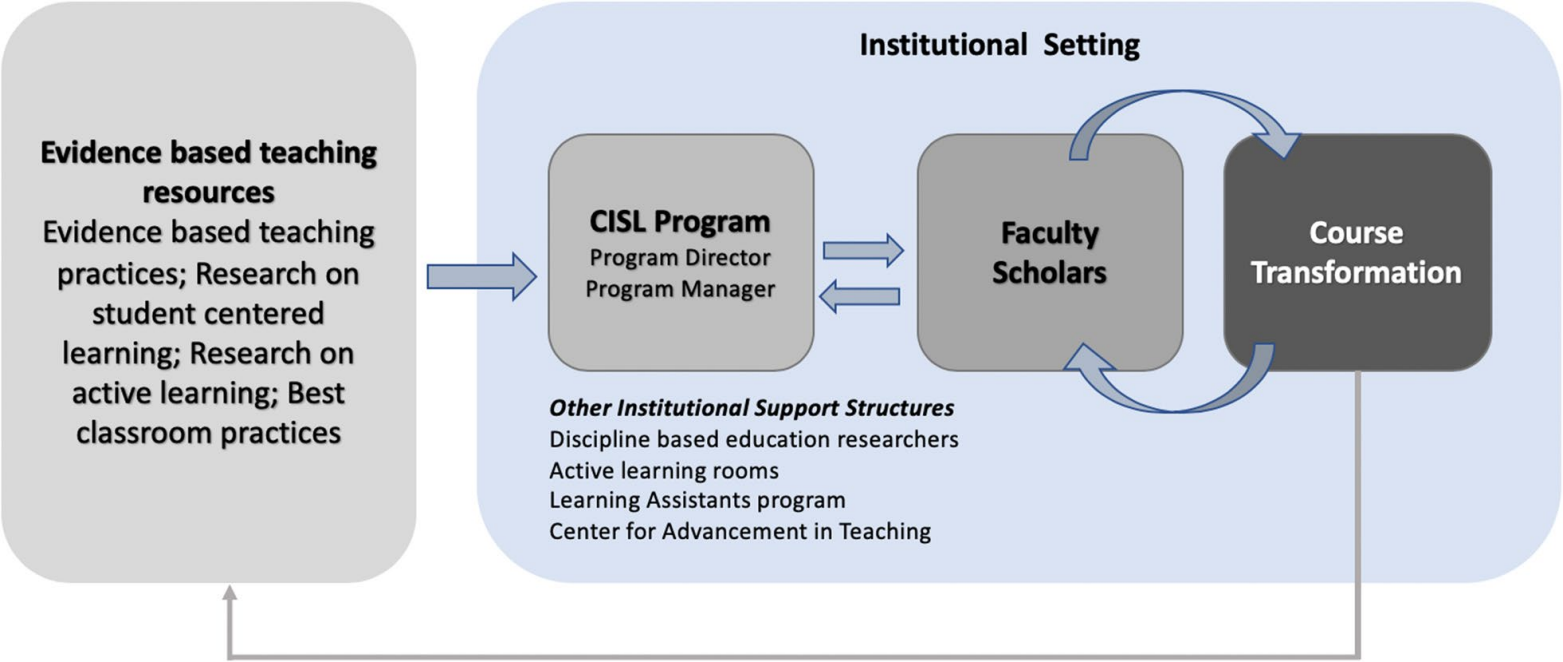
Overview of the CISL Faculty Scholar program. The CISL program is one of the institutional support structures that serves as the communication link between the resources on evidence-based teaching practices and Faculty Scholars. There is regular feedback (arrows) between CISL program and Faculty Scholars. Faculty Scholars implement course transformations which in turn provides feedback that is incorporated into the next iteration of course transformation as indicated by the rounded arrows. Course transformations by Faculty Scholars can contribute through peer-reviewed publications (grey arrow)

In this study, we describe a program, Collaborative for Institutionalizing Scientific Learning (CISL), which provided faculty members in a large research-intensive Hispanic Serving Institution with the training and the support structures to bring about individual-level changes and lead to increased number of faculty using evidence-based teaching practices. The CISL program acts as a communication link between resources (e.g., evidence-based teaching practices) and individual STEM faculty members to support them in the implementation of research-based instruction in their courses, by following an iterative feedback mechanism (Fig. 1). The CISL program followed the underlying logic that STEM undergraduate instruction will be changed by not only developing research-based instructional “best practices” but also training and supporting instructors to use them (Borrego & Henderson, 2014).

### CISL Faculty Scholar program

The CISL Faculty Scholar Program operated in a 1-year cycle, where Faculty Scholars were selected to reform a course of their choosing (i.e., *Application Stage*); they worked with a program Assistant Director to design and prepare for their course transformation (i.e., *Design Stage*); and implemented and assessed their course transformation over the course of two semesters with support from the Assistant Director and undergraduate Learning Assistants (i.e., *Implementation Stage*). During the 1-year cycle of the program, the Assistant Director worked individually with Faculty Scholars from the cohort and supported them through the design and implementation stages. The CISL program also provided continued support after the implementation stage, in the form of data analysis and community development.

For the CISL *Application Stage*, faculty members from science and mathematics departments applied to be a Faculty Scholar and proposed a transformation of a STEM course of their choice. The main intention behind initiating a call for application was to provide faculty members the opportunity to reflect on the need for transformation in their classrooms and provide them agency over the decision to transform their courses. A call for application was sent out to all faculty members from participating science and mathematics departments every year in early spring, disseminated by Department Chairs, program Co-Directors, and faculty listservs. In late spring, a committee of the program leadership team (i.e., CISL program Director, Co-Directors representing each of the participating departments, and the Assistant Director) selected a cohort of applicants based on their application materials. Faculty Scholars were competitively selected based on their readiness for course reform and the potential impact of their course transformation on student success and departmental change.

During the *Design Stage*, Faculty Scholars worked closely with the CISL Assistant Director, who served as a communication link between evidence-based teaching resources and the faculty members. More specifically, the Assistant Director worked individually with every Faculty Scholar during the summer to help them refine the student goals and expectations for their course using Fink’s model of course redesign to create significant learning experiences (Fink, 2013). At this stage, the Assistant Director primarily communicated the vision of effective teaching to Faculty Scholars and introduced them to evidence-based teaching practices, research on student-centered learning and active learning, and new ways to organize or teach a subject (i.e., curriculum and pedagogy). The design stage started with several individual meetings during which the Assistant Director and Faculty Scholar in that year’s cohort discussed the general structure of their course, their motivations to transform the course and CISL program support and expectations. The main intention behind these initial meetings was to identify specific areas where transformation efforts could be focused for each Scholar and develop a plan for course redesign. Once specific areas were identified, the Faculty Scholar spent the next part of this stage developing materials and lesson plans to incorporate specific evidence-based instructional strategies into their courses. The Assistant Director’s main role at this time consisted of connecting the Faculty Scholars with relevant evidence-based resources; providing feedback on course redesign and working with the Scholars in creating significant learning activities for their courses. As the Assistant Director worked individually with each Faculty Scholar, the type of support provided varied across Scholars and depended on their specific focus for course transformation and their experience with evidence-based teaching practices. The design stage was generally conducted over a summer semester and Faculty Scholars were provided 3 weeks of summer salary to protect their time from other duties. During this stage, the Assistant Director met with Faculty Scholars at least once every week.

Faculty Scholars then implemented their course redesign over 2 consecutive semesters (i.e., *Implementation Stage*) and collected data to assess the effectiveness of the transformation. The main objective of this phase was to implement and assess the course reform and solidify the use and practice of evidence-based teaching strategies in Faculty Scholars’ classrooms. A key element of the implementation is that all course transformations capitalized on an existing Learning Assistant (LA) program and incorporated undergraduate LAs during class time to facilitate student discussions and engagement. During this phase, the Assistant Director individually met with each Faculty Scholar from that year’s cohort on a weekly basis to discuss the day-to-day management of their transformed course. Weekly meetings mainly revolved around (but were not limited to) discussing student buy-in of course transformation; mitigating challenges with transformed course elements; reflecting and incorporating feedback provided by the LAs; reflecting on student performances on quizzes, midterms and other assignments; and developing a plan for evaluation of transformed course elements. The Assistant Director also performed classroom observations of the Faculty Scholars’ courses about 1–2 times during the semester to provide detailed feedback. Classroom observations were generally scheduled to be once at the beginning of the semester and once towards the end of the semester.

Learning Assistants (LAs) were a key element of the implementation phase. The LA model is a form of near-peer instruction designed to support changes towards evidence-based instruction as well as promote shifts in attitudes among students, faculty, and administrators (Barrasso & Spilios, 2021; Kornreich-Leshem et al., 2022). LAs are undergraduate students that have both disciplinary and pedagogical knowledge and are, thus, able to facilitate collaborative peer learning during group work in class while also supporting faculty change towards evidence-based teaching (Goertzen et al., 2011; Otero et al., 2006). The LA program has three fundamental components that the LAs participate in: (1) a semester-long pedagogy course to learn about teaching, reflect on their experiences, and get support from fellow LAs; (2) weekly preparatory meetings with faculty instructors to reflect on students’ progress, discuss course content, plan for upcoming lesson, and provide feedback to instructors on the student perspective; and (3) the actual practice of supporting implementation of evidence-based teaching practices through facilitating learning within a classroom and guiding students in their own learning (Barrasso & Spilios, 2021; Kornreich-Leshem et al., 2022). Importantly for the CISL program, LAs can provide feedback to faculty from a students’ perspective both related to the course content and the climate of the course, thus strengthening the faculty member’s understanding of their students (Davenport et al., 2018).

An important objective at the implementation phase is also to collect student measures to provide evidence of effectiveness. Faculty Scholars were supported by the CISL program to find and use instruments and protocols developed by the STEM education research community, when available, and collect student metrics in their courses. Faculty Scholars were supported through the collection, analysis, and interpretation of course data to aid in their course reflection and potentially lead to publishable results. Generation of publication-worthy evidence was a means by which Faculty Scholars contributed towards the body of research-based instructional practices and further reflected on their own practices (Fig. 1). Financial support for faculty was provided in the form of overload salary for two implementation semesters, financial support to hire LAs, and course-buy outs, when appropriate.

Faculty Scholars who had completed the 1-year program cycle continued to be supported by the Assistant Director. After the implementation stage, the Assistant Director supported these Faculty Scholars through collection, analysis, interpretation, and dissemination of course data. Faculty Scholars also had access to the program financial support in the form of travel awards to education-relevant conferences or summer research supplements to support further data analysis and manuscript preparation. Finally, Faculty Scholars became part of a growing community of active learning adopters that shared practices and knowledge.

## Research questions

In this study, we are interested in assessing the impacts of the CISL program on both Faculty Scholars and student performance. We are also interested in identifying specific elements of the program that provided maximum support to Faculty Scholars, as perceived by them. To guide us in our data collection and analyses, we addressed the following research questions: (1) What factors motivated faculty and instructors to apply to the CISL Faculty Scholar program and reform a course they are teaching?; (2) To what extent did CISL Faculty Scholars use evidence-based teaching practices and how did it impact student performance and success?; (3) What elements of the CISL program best supported Faculty Scholar’s course transformations and how? What programmatic supports were identified to be critical to sustaining course transformations?

## Methods

We evaluated the impact of the CISL program through a mixed-methods approach incorporating multiple outcomes. Metrics included investigating the impact of the program at the student-, Faculty Scholar-, and institutional-levels, as measures of effectiveness. Data for our study came from various sources (see Table 1 for all data sources and collection timepoints). Methods below are organized by Research Question. This study was approved by the university’s Institutional Review Board (IRB) as an exempt research protocol.

**Table 1:** Summary of the data collection for each research question

Research question	Data source	Collection timeline and observations
RQ1	Faculty Scholar application materials	30 applications (28 individual, 2 group applications)—all cohorts (2012–2021)
RQ2	Faculty Scholar COPUS classroom observations	14 Faculty Scholars observed more than one time (cohorts 2013–2019) during first year of implementation
	ChIPP survey	16 Faculty Scholar respondents (cohorts 2012–2016)—during or after implementation phase
	Student institutional data	Data from students in Faculty Scholars CISL course before and after course transformation (Fall 2008–Spring 2021)
	Faculty Scholars interviews	14 Faculty Scholar interviews (2011–2019 cohorts)*
RQ3	Faculty Scholars interviews	*Same as RQ2
	Administrator interviews	10 administrator interviews (8 in 2015, and 2 in 2018)
	Survey administered to Faculty Scholars	39 Faculty Scholars respondents, all cohorts (2012–2021)

### Faculty data set

41 Faculty Scholars were supported through the CISL program from 2011 to 2019. The demographics of the Faculty Scholars included 44% male, 27% racial/ethnic minority, 54% tenure-track research faculty, and 46% instructional faculty. The Faculty Scholars included faculty from the following departments: Biology (*n* = 14), Chemistry (*n* = 9), Physics (*n* = 4), and Mathematics (*n* = 14). Faculty Scholars represented 19%, 20%, 13%, and 21% of total active full-time research and instructional faculty in the departments of Biology, Physics, Chemistry, and Mathematics, respectively. Reformed courses included courses at the introductory-level (50%, *n* = 14) and upper-division (50%, *n* = 14); with most courses being the lecture-part of the course (93%, *n* = 26) and a few laboratory (7%, *n* = 2). Almost all course transformation efforts included the implementation of partially to fully inverted classrooms (DeLozier & Rhodes, 2017) and use of Learning Assistants during class time (Otero et al., 2006). Of the courses transformed, three courses were transformed by two or more Faculty Scholars working as a team.

### Research question 1: what factors motivated faculty and instructors to apply to the CISL Faculty Scholar program and reform a course they are teaching?

#### Application materials

We examined application materials of awarded CISL program applicants to investigate faculty motivation. On average, the program received 7 applications per year (min 4, max 10) and awarded approximately 4 per cycle (min 2, max 6). Over the 8 years, 30 applications comprising 41 Faculty Scholars were awarded and supported by the CISL program (2 awarded applications consisted of a group of 5 and 8 faculty members, respectively); out of a total of 61 total applications received (54 individual and 7 group applications). To analyze application materials of Faculty Scholars, we conducted thematic analyses of responses to the open-ended question: “What is your motivation for transforming this course?” Two researchers initially coded the faculty responses to this question, individually. After this initial round of coding, the codes were compared and discussed, to generate a list of codes and definitions. In subsequent coding, the researchers coded individually, compared their codes, and then reached consensus through discussion of instances in which their codes initially disagreed. Interrater reliability was substantial with Cohen’s kappa *K* = 0.68. Codes were combined into larger themes, through collaborative discussions between the coders, to arrive to the final themes (Table 2). Please note that Faculty Scholars self-select to be part of the program and, thus, these results may not extend to all faculty, particularly those that are disinclined to adopting evidence-based strategies.

**Table 2:** Summary of qualitative themes of Faculty Scholar’s motivation to transform their course (at application stage)

Subcategories	Definition
Faculty centered motivations	
Faculty dissatisfaction	Faculty is dissatisfied by current state of the course, by their ineffective use of instructional strategies, or by the lack of student learning
Faculty enthusiasm	Faculty shows enthusiasm for implementing active learning and for its promise of improving student outcomes and teaching implementation
Faculty influenced by positive experience with active learning in the past	Faculty has piloted innovative teaching strategies and has had a positive experience (e.g., positive student outcomes, student evaluations, or instructor benefit/ enjoyment)
Faculty influenced by professional development events	Faculty participation in professional development workshop/event informed their decision of seeking innovative teaching strategies
Faculty influenced by interaction with colleagues	Faculty interactions with peers (i.e., faculty colleagues) that have been implementing innovative strategies informed their decision to reform the course
Student centered motivations	
Faculty perception of deficiencies in students	Faculty negative perception of students' preparation (e.g., lack of pre-requisite content knowledge and/or skills) informed decision to reform the course and better support students
Opportunity to develop student skills	Course transformation will provide an opportunity to develop key skills (e.g., critical thinking, problem solving and communication skills) that promote student success in major
Opportunity to increase student learning	Course transformation will provide an opportunity to increase student learning and understanding of course materials
Opportunity to increase student engagement/attitudes towards course/discipline	Course transformation will provide an opportunity to increase student engagement and/or positive attitudes (e.g., motivation, value, enjoyment) with the course material
Opportunity to enhance student satisfaction	Course transformation will provide an opportunity to enhance student satisfaction with the course, sometimes by addressing concerns voiced by students
Course centered motivations	
Challenges faced due to the nature of the course content	Course difficulty is high due to content nature or amount, or the perceptions students have about the course
Challenges faced due to the high enrollment in the course	Course’s large enrollment makes it difficult to engage students without additional modification and help (e.g., Learning Assistants)
Low student outcomes in the course	Student outcomes are low in this course (e.g., low passing rates that influence STEM major retention)
Opportunity to improve course structure	Course transformation will improve the structure and better support student success (e.g., engagement, skills, etc.)
Course is important for student’s career or degree success	Course is important for students either because the content or skills are required for successfully completing current degree, the content might be included in future career or career related examinations, or the course helps students be more well-rounded individuals
Department centered motivations	
Opportunity to promote change in department	Transforming the course will promote and/or sustain change in the department (e.g., develop materials that others can use, set a precedent, benefit an ongoing change such the creation of a major)
Opportunity to improve department metrics	Implementing active learning will help improve departmental/institutional metrics (e.g., graduation, retention, etc.)

### Research question 2: to what extent did CISL Faculty Scholars use evidence-based teaching practices? How did Faculty Scholar participation in the CISL Program impact student performance and success?

#### Interviews

The external evaluator, WestEd, conducted semi-structured interviews of Faculty Scholars and key administrators at two timepoints. The first round of interviews of a random sample of Faculty Scholars (*n* = 7 out of 25 active Faculty Scholars) and administrators (*n* = 8) was in August 2015; followed by a second round of interviews of Faculty Scholars (*n* = 7 out of 41 active Faculty Scholars) and administrators (*n* = 2) in August 2018 (total; 14 Faculty Scholars and 10 administrators). Participation in the interview was voluntary and anonymous to CISL program members. Please see Additional file 3 for interview protocol.

#### Survey of faculty perception of their instructional practice use

**Fig. 2:**
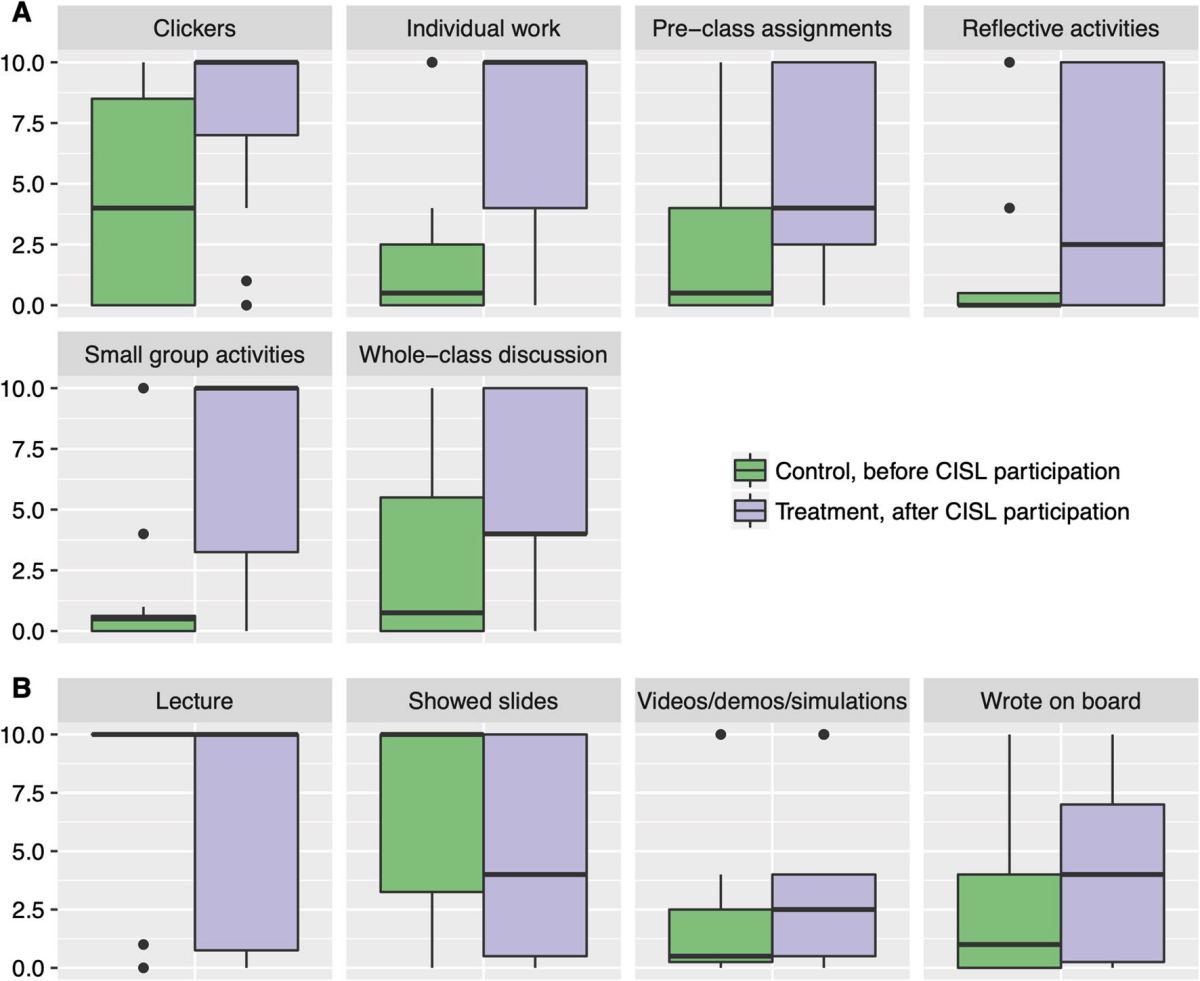
Frequency of use of instructional practices by Faculty Scholars before and after CISL program participation. This figure shows frequency of use of student-centered (**A**) and instructor-centered (**B**) instructional practices for a sample of Faculty Scholars, before or after participation in the CISL program. The faculty reported their frequency of use of instructional practices in the STEM course they most frequently teach either in the most recent occasion they taught the course (i.e., treatment, after CISL participation, in purple) or in the oldest occasion in the last 5 years (i.e., control, before CISL participation, in green). The boxplots show the distribution of responses, with the median indicated by the middle horizontal line, the box representing the middle 50% of scores for the population, and the upper and lower whiskers indicating the range of the upper and lower quartile, respectively. Please note that items with highly skewed responses (e.g., Lecture, control) will have compressed boxplots. The *y*-axis is a linearized scale of the frequency of use of instructional practices in a term, using the monthly frequency as the unit with 0 = ”Never”, 0.5 = “1–2 times per term”, 1 = “Monthly”, 4 = “Weekly”, and 10 = “Every class”

We analyzed data from the Change in Implementation of Pedagogical Practices (ChIPP) survey, a national-level survey of teaching practices collected in 2016, which included CISL Faculty Scholar responses (Benabentos et al., 2020). The national sample included biology, chemistry, and physics faculty across research-intensive institutions that had been awarded grants from Howard Hughes Medical Institute (HHMI), through their 2014 Science Education grants for Research Universities program (*n* = 1456 faculty members from 31 institutions). Briefly, faculty in biology, chemistry, and physics departments were recruited to respond to the survey anonymously, which included questions on frequency of use of instructional practices of their most commonly taught STEM course. Only responses from faculty that indicated participation in the CISL’s Institutional “HHMI Science Education Program” were included in this analysis, as Faculty Scholars that have implemented their course reform (out of 25 CISL Faculty Scholars in 2016, 16 or 64% responded to this survey). In the ChIPP survey, faculty were asked to report on how frequently they used particular instructional practices at two points in time: (1) in the most recent occasion they taught it and (2) in the oldest occasion in the last 5 years. Because of the time of survey administration, this data set allows us to assess changes in the self-reported instructional practices of Faculty Scholars before and after CISL participation. Faculty indicated the frequency of use of instructional practices on a 5-point frequency scale with response choices “Never”, “1–2 times per term”, “Monthly”, “Weekly”, and “Every class”; both in the most recent occasion they taught the course and in the oldest occasion they taught the course (within the last 5 years). We are reporting on Faculty Scholar responses to 10 commonly used instructional practices (Fig. 2, Additional file 1: Table S1). Instructor-centered instructional practices reported include (1) lecturing, (2) showing slides (e.g., PowerPoint), (3) writing or solving problems on a chalk or white board, and (4) showing videos, simulations or demonstrations related to the material. Student-centered instructional practices reported included (1) collecting responses from students in real-time (e.g., clickers), (2) holding whole-class discussions, (3) having students do small group activities, (4) having students do individual work in class, (5) giving students assignments or quizzes on the readings/videos prior to covering material in class (i.e., pre-class assignments), and (6) asking students to engage in reflective activities (e.g., 1-min paper, think-pair-share). It is important to note that, since ChIPP relies on participant self-reported data, a limitation is that the survey responses represent faculty’s perceptions and might not correspond to their actual use of instructional practices. In addition, only 16 Faculty Scholars (out of a total of 41 potential participants, 39%) completed the survey and this group may not be representative of all Faculty Scholars’ potential responses.

#### Classroom observations

Two researchers conducted classroom observations using an adaption of the Classroom Observation Protocol for Undergraduate STEM (COPUS) (Smith et al., 2013). Whole class periods of Faculty Scholars were observed (minimum class period is 50 min) and instructor actions were coded every 2 min. Instructional practices that were instructor-centered were grouped together and included the following codes (Additional file 1: Table S1): “Lecturing”, “real-time writing on the board, document projector, etc.”, and “showing or conducting a demo, experiment, simulation, video or animation”. Instructional practices that were student-centered were grouped together and included the following codes: “asking clicker questions”, “follow-up discussion after clicker question”, “posing non-clicker questions to all students (non-rhetorical)”, “listening to and answering student questions with the entire class listening”, “moving through class and looking at student work”, “one-on-one discussions with an individual student” or “talking/interacting with a group of students”. Student-centered instructor behaviors occurred when students were usually working on activities either in small groups or individually, during class. Additional instructor behaviors that didn’t fit either grouping were coded as “Other” (e.g., making announcements, returning quizzes, waiting at the beginning of class). The total percentage of time spent in instructor-centered or student-centered instructional practices during a class period was calculated by adding the number of 2-min intervals that included either instructor- or student-centered behaviors and dividing by the number of total 2-min instances in the class period. It is important to note that, even though overlap was not common, it is possible for an instructor to engage in both instructor and student-centered behaviors in the same 2-min interval. The sample included a total of 14 Faculty Scholars who were observed by an individual researcher between 2 and 7 times (average 2 times) in their 1st year of implementation between 2014 and 2020. For each Faculty Scholar observed, we calculated the average percent time spent in instructor- and student-centered behaviors across all their observations. From these averages, we calculated an average of all Faculty Scholars (Table 3). Some methodological limitations to be noted include: (1) Only 14 Faculty Scholars (out of a total of 41 potential participants, 33%) were observed more than once during their 1st year of implementation using the COPUS methodology. This group may not be representative of all Faculty Scholars’ teaching practices. (2) As classroom observations did not include any data collection before participation in the CISL program, this analysis does not provide evidence of change in use of evidence-based teaching practices after participation in the CISL program. (3) Even though instructor-centered approaches would usually involve direct instruction (e.g., lecturing with students taking notes), these approaches may include strategies that actively engage students (e.g., working a problem together on the board, actively soliciting students’ questions).

**Table 3:** Faculty Scholar percent time on instructor- and student-centered instructional practices, per class session

Metric	Percentage
Instructor-centered instructional practices	
Average time per class session	46.18% (± 17.65%)
Minimum time per class session	13.16%
Maximum time per class session	79.75%
Student-centered instructional practices	
Average time per class session	63.65% (± 12.99%)
Minimum time per class session	47.90%
Maximum time per class session	92.11%
Total FS with 2 or more COPUS observations	33.33% (14/41)

#### Student quantitative data set

To investigate impact on student performance, we collected data on student outcomes from the courses that were redesigned and taught by Faculty Scholars (see Additional file 1: Table S2), including historical data before Faculty Scholars CISL program participation (Fall 2008–Spring 2021, excluding summers). For each Faculty Scholar, we only included the student data for the course they chose to redesign, either before or after program participation (i.e., non-CISL and CISL-treatment conditions, respectively). We removed any other assigned courses the Faculty Scholars taught during the semesters, from analysis. Course Grade Point Unit (i.e., the numerical course grade in a 4.0 scale, GPU) was used as the indicator of student performance. Student data points with grades that did not impact the GPA (e.g., audit, pass/fail) were removed from the analysis (*n* = 1247). The data file included 65,175 records with 33,543 unique students and 40 Faculty Scholar instructors. The sample variables included STEM major status (66% of the observations were STEM majors), sex (40% male students, 3.1% missing data), transfer status (35% students transferring from 2-year institutions, 4.3% missing data), race/ethnicity minority status (83% students identify with race/ethnicity groups minoritized in STEM fields, 3.1% missing data), STEM/non-STEM major type (67% of students were had declared STEM majors at the time of taking the course), high-school GPA (average 3.8/4.0, ± 0.64 SD, 8.3% missing data).

#### Statistical analyses

To compare student outcomes, we employed a multiple regression analysis, using GPU as the outcome variable. The treatment was defined as any course that was reformed through the support of the CISL program and (1) had an instructor who had been supported as a CISL Faculty Scholar; and (2) had Learning Assistant support in the course. The regression model included variables controlling for student prior academic performance (i.e., high-school GPA), STEM major status, sex, race/ethnicity minority status, and transfer student status. We included these variables, because they have been documented to be common predictors of students’ course performance, attributed to systemic and structural biases experienced by those groups (Allensworth & Clark, 2020; Eddy & Brownell, 2016; Lakin & Elliott, 2016; Theobald et al., 2020). Effect sizes between means was calculated using Cohen’s *d*. Average passing rates was calculated by calculating the percent of students being awarded a C grade (GPU = 2.0) or higher in a group and dividing by the total number of students in the group. Percent change in passing rate was calculated using ((*V*_2_ – *V*_1_)/*V*_1_) × 100, in which *V*_2_ represents the treatment passing rate and *V*_1_ represents the control passing rate (i.e., historical data before CISL participation). Analyses were performed in R (R Core Team, 2017), the open-source programming and software environment for statistical computing and graphics. Throughout these analyses, we set alpha, the maximum acceptable chance of Type I error, to 1%. One limitation of this analysis is that it is using academic performance as the sole outcome of student success and may miss other important student measures, such as socio-cognitive outcomes.

### Research Question 3: what elements of the CISL program best supported Faculty Scholar’s course transformations and how? What programmatic supports were identified to be critical to sustaining course transformations?

#### Interviews

Semi-structured interviews of Faculty Scholars and key administrators were conducted at two timepoints, as described in the methods under Research Question 2.

#### Survey on programmatic impact

**Fig. 3:**
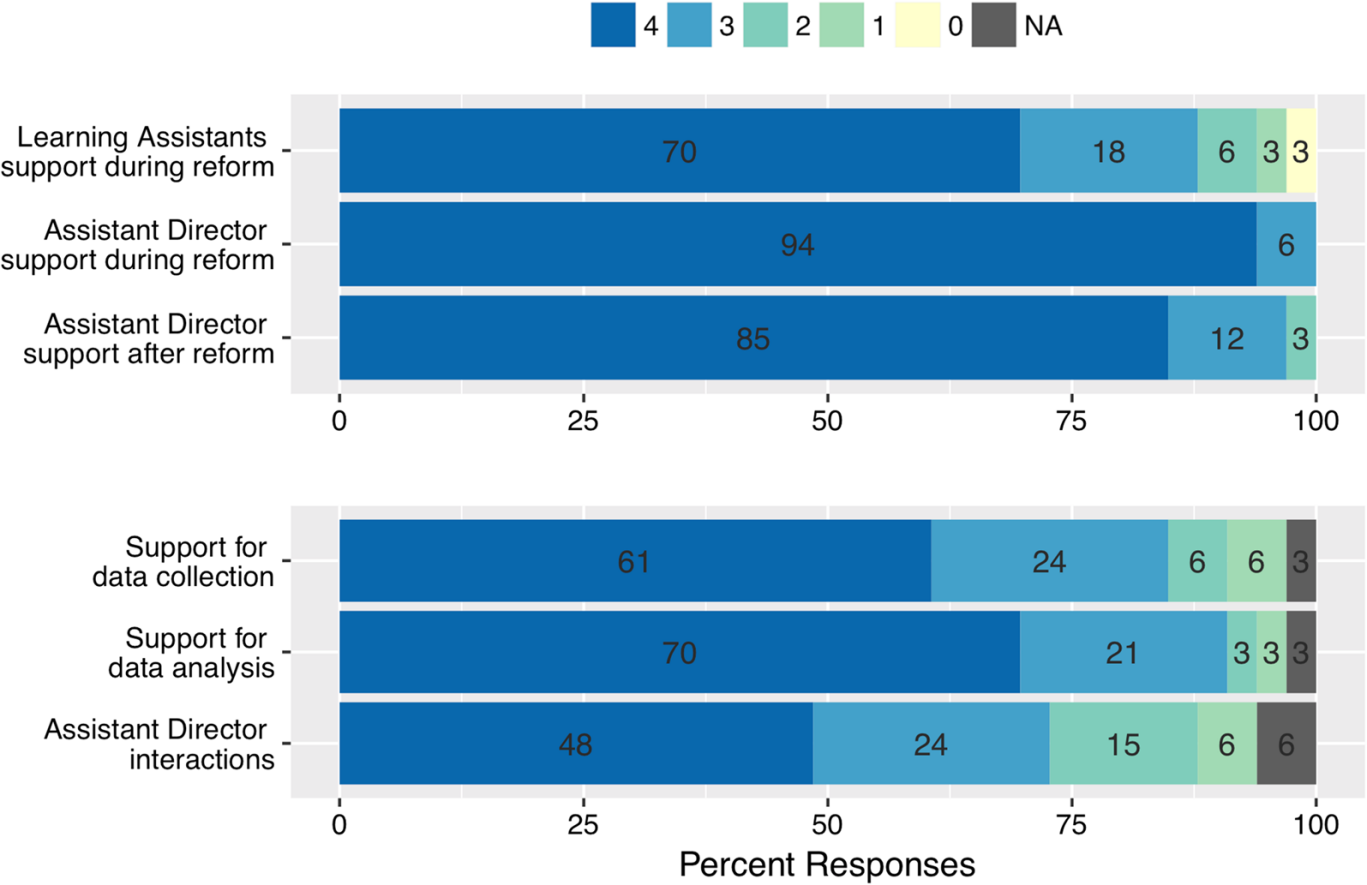
CISL Personnel Program Support. Percent of Faculty Scholars reporting their perceptions on the extent of the benefit of certain programmatic elements stemming from the Assistant Director or Learning Assistant support. Faculty responses were recorded on an anchored 5-point rating scale, with rating 4 indicating highest importance (i.e., “very important”) and rating 0 indicating lowest importance (i.e., “not at all important”). Numbers superimposed in the bars are the percentage of respondents choosing that option (*N* = 33)

**Fig. 4:**
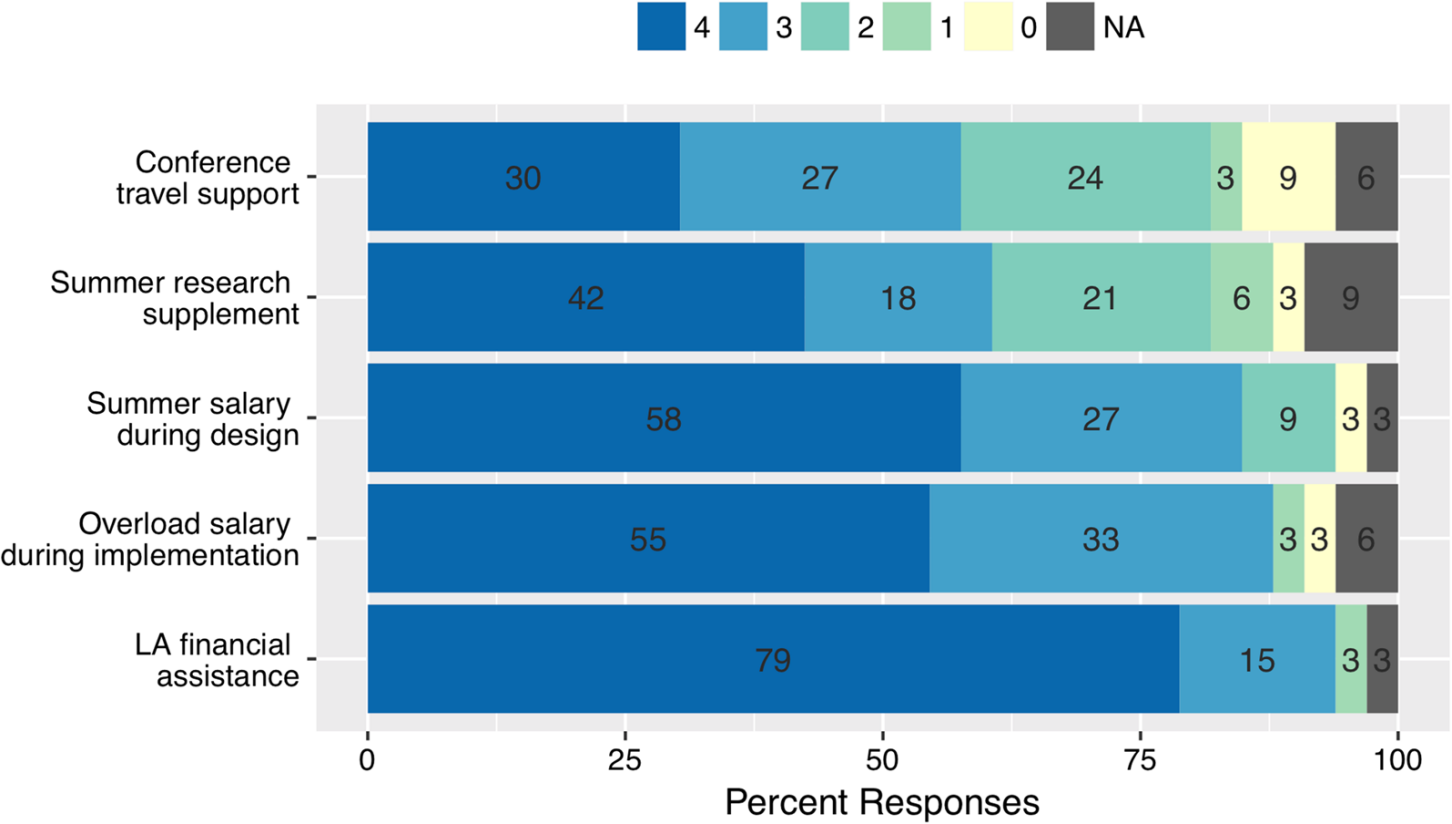
CISL Program Financial Support. Percent of Faculty Scholars reporting their perceptions on the extent of the benefit of certain programmatic elements that provided financial support. Faculty responses were recorded on an anchored 5-point rating scale, with rating 4 indicating highest agreement (i.e., “very important”) and rating 0 indicating lowest importance (i.e., “not at all important”). Numbers superimposed in the bars are the percentage of respondents choosing that option (*N* = 33)

**Fig. 5:**
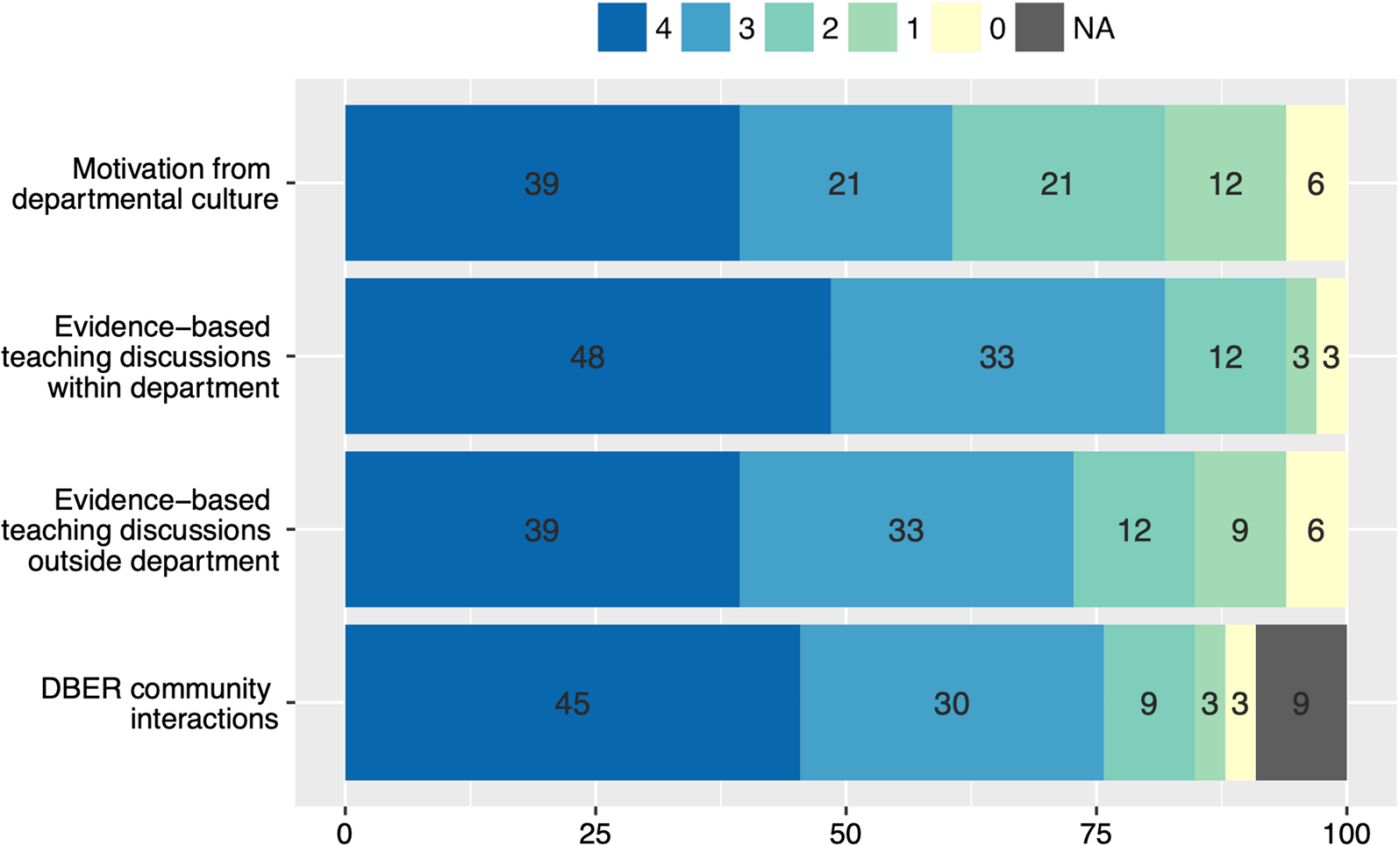
Community. Percent of Faculty Scholars reporting their level of engagement with departmental and institutional faculty communities. Faculty responses were recorded on an anchored 5-point rating scale, with rating 4 indicating highest importance (i.e., “very much so”) and rating 0 indicating lowest agreement (i.e., “not at all”). Numbers superimposed in the bars are the percentage of respondents choosing that option (*N* = 33)

WestEd administered an anonymous 16-item survey in Spring 2020 to all Faculty Scholars who had been supported through the CISL program in transforming their courses between 2011 and 2019. The survey instrument was informed by the responses from the interviews conducted in August 2015 and 2018. The survey asked Faculty Scholars general questions about themselves and their course transformation as well as their perceptions of the CISL program and its supports, through a combination of multiple choice and open-ended questions. Participation in the survey was voluntary and anonymous to CISL program members. The response rate to the survey was 93% (*n* = 39 out 41 Faculty Scholars), with the majority of respondents being non-tenure-track teaching faculty (48%), followed by tenured faculty (43%), and then pre-tenure faculty (9%). In the survey, Faculty Scholars were asked to rate how important were elements of the program in supporting their transformation efforts and sustaining the changes. Elements in the survey included personnel support (e.g., Learning Assistants, CISL Assistant Director), financial support (e.g., summer salary), and community engagement (e.g., support from Discipline-Based Education Research faculty). Faculty responses to personnel and financial support (Figs. 3 and 4) were recorded on an anchored 5-point rating scale, with rating 4 indicating highest importance (i.e., “very important”) and rating 0 indicating lowest importance (i.e., “not at all important”). Faculty responses to departmental engagement (Fig. 5) were recorded on an anchored 5-point rating scale, with rating 4 indicating highest agreement (i.e., “very much so”) and rating 0 indicating lowest agreement (“not at all”). A list of the survey items is included in Additional file 1: Table S1.

## Results

### Research Question 1: faculty are motivated to apply to the CISL program and reform a course due to faculty-, student-, course-, and departmental-level motivations

Faculty Scholars were asked to answer the open-ended question: “What is your motivation for transforming this course?” in the application materials for the CISL program. Analysis of the responses of awarded Faculty Scholars revealed four broad categories of motivation to transform their proposed courses (Table 2): (1) *Faculty-centered motivation*, in which faculty are motivated by their personal knowledge, experiences, beliefs and attitudes; (2) *Student-centered motivation*, in which faculty are motivated by the need to improve student performance in the proposed course or for future courses; (3) *Course-centered motivation*, in which faculty are motivated to improve course structure, because course content is difficult and/or important for student success; and (4) *Department-centered motivation*, in which faculty are motivated to make department level impacts through course transformations. Faculty Scholars tended to mention a combination of these categories in their application. Within these four main categories, we further identified subcategories to investigate the specific motivations influencing faculty to apply to the CISL program (Table 2). In general, most faculty displayed more than one main category of motivation and several sub-categories of motivation.

#### Faculty-centered motivation

Faculty motivation to transform a course stemmed in part from Faculty Scholars’ beliefs, attitudes and personal experiences with active learning, which led to enthusiasm about the potential positive outcomes of course reform. A considerable number of Faculty Scholars displayed dissatisfaction by the current state of their course and believed transforming their courses would be the way to address their concerns:

*“I lecture interactively and we solve problems in class but try as I might I cannot get to everyone. During these sessions, I see students working with each other and helping each other. However, my facilitation leaves a lot of be desired, because it is one lecturer for 200 students.”*

Balancing this sentiment, there was also evidence of Faculty Scholars’ enthusiasm in implementing active learning strategies, due to its promise of improving student outcomes and teaching implementation and faculty’s own positive beliefs on these practices. This motivation is evident from these sample responses by Faculty Scholars showing positive attitudes towards active learning and their hope that these practices would help students’ learning experiences:

*“Active learning in the form of clicker questions is phenomenal for lectures as it breaks the monotony of the lecture, opens a space for thinking, and lets the instructor know if the concepts are coming across”*

*“I feel students and myself are missing the opportunity of really gaining a unique learning experience by incorporating activities requiring more engagement on their end. Based on that, I would like to incorporate more learning experiences that actually make students think about what they are doing and make the learning experience a group experience, rather than a sum of individual efforts.”*

Several Faculty Scholars were also found to be influenced by past positive experiences in which they had piloted active learning practices in their own classrooms. These positive experiences included encouraging student feedback and evaluations (i.e., *“[piloting active learning strategies was] an amazing experience, highly positive for students, as indicated in their course evaluations”*), as well as benefits to student learning and faculty insight into areas which students might be struggling with. Several Faculty Scholars also commented on the positive influence of external sources, such as interacting with other faculty members that have implemented active learning successfully and/or by participating in professional development workshops or events related to active learning, as an encouraging motivation to pursue reforming their course. These experiences fueled Faculty Scholar’s intrinsic motivations to participate in course transformations.

#### Student-centered motivations

Faculty Scholars were also found to be motivated by the potential of impacting students’ learning and future success, an intrinsic motivation focused on faculty’s intentions to provide opportunities for student learning. Some of the motivations spur from faculty’s negative perceptions of students’ preparation for their course, as they perceive students lack pre-requisite content knowledge and/or skills needed to succeed in the course (i.e., deficiency in students, Table 2). Thus, they are motivated to transform the course to better support students that have this perceived lack of preparation. Faculty’s perception of students’ deficiencies focused on perceived ineffective study strategies and perceived lack of content knowledge:

*“[Students] lack the critical thinking and problem-solving skills needed to do well in [this course], primarily because the focus in their previous classes has always been on rote memorization”*

*“Many students come to this course having obtained passing to higher grades in the prerequisites, yet unable to use these problem-solving abilities. I attribute this to these students’ ability to study just enough to get through the prerequisites with shallow (soon to evaporate) knowledge”*

The other four sub-categories (opportunity to develop student skills; opportunity to increase student learning; opportunity to increase student engagement/attitude towards course/discipline; opportunity to enhance student satisfaction; Table 2) encapsulate the different areas that Faculty Scholars believe transforming their course would help improve student skills and learning. For example, a Faculty Scholar believed that transforming the course would provide an opportunity to develop student skills, while another respondent was motivated by the opportunity to increase student engagement and/or attitudes towards the course/discipline:

*“Students are expected not only to learn [the course] concepts, but also develop critical thinking and analytical skills, which would help them in solving theoretical and practical [course related] problems. I believe that active learning is the best way to engage students and help them to develop required skills.”*

*“It is also our hope that this more engaging style of instruction will give students greater comfort with and enjoyment of [the discipline] and that this more positive attitude towards quantitative ideas will apply to all their work in STEM fields.”*

#### Course-centered motivations

Faculty Scholars’ responses also highlighted course-centered motivations related to course characteristics faculty perceived as challenging and, therefore, provided the motivation to transform the course. One common feature identified to be challenging to the Faculty Scholars was the nature of the course content (i.e., challenges faced due to nature of the course content, Table 2):

*“[The] exercises are time consuming. Students have difficulty carrying out these manipulations properly. Due to time constraints, I can only do a few exercises with them. Usually some students catch on very quickly and are able to progress through the course well. Other students have a really difficult time doing this”*

This Faculty Scholar believed that transforming the course will give them an opportunity to address the inherent challenges of the course content. Another common challenging characteristic recognized by Faculty Scholars was that their courses were high enrollment, making it difficult for them to engage students without additional modification and help (i.e., challenges faced due to high student enrollment in the course, Table 2) as evident from the response of one Faculty Scholar facing this challenge: *“More than one student has commented on how they felt lost in the 200-person lecture hall”*.

Another course level challenge faced by some of the Faculty Scholars was low course outcomes, such as a high failure rate or low student retention in the major. A significant proportion of Faculty Scholars considered their courses to be important for students’ success (i.e., course is important for students’ career or degree success, Table 2) and hence, were motivated to transform them. For example, one Faculty Scholar commented that *“[their course] is one of the gateway courses and students’ success in it is critical in the pursuit of their STEM or Business degree”*.

Similarly, a considerable proportion of Faculty Scholars also highlighted that transforming the course would provide them with an opportunity to improve the current course structure and provide more support to students: For example, a Faculty Scholar wrote: *“I would like to transform this course in such a way that students have more opportunities for guided learning either *via* the use of Learning Assistants or the flipped classroom concept”.*

#### Department-centered motivations

Finally, some Faculty Scholars were motivated to impact their department through transforming their courses, as they believed that transforming their courses would promote and/or sustain change in the department. Examples of ways to promote departmental change was developing teaching resources that others can use or setting a precedent on how a course could be taught (Table 2):

*“After this course is implemented and successfully taught, it will help set a precedent in the department; [I] hope it will encourage other faculty to update their obsolete methodologies and innovate and follow similar methodologies to the one proposed here”*

In addition to this sentiment, some Faculty Scholars were also motivated to improve department metrics through transforming their courses, as evident from this Faculty Scholar’s response: *“Adding this [reform] will help the department meet both these metrics [graduation rate and employment within 1 year of graduation] in a more efficient and effective way”*.

### Research Question 2: evidence-based instructional practices after participation in the CISL Faculty Scholar program and its impact on student performance and success

#### Faculty Scholars’ self-reported change in instructional practices

Faculty Scholar responses to the ChIPP national survey (Benabentos et al., 2020) self-reported an increase in the frequency of use of student-centered instruction in their reformed course. ChIPP collected information on the frequency of use of instructor- and student-centered teaching strategies for Faculty Scholars in the program as of 2016 (including both first-time and experienced Faculty Scholars). CISL faculty participants that responded to the survey (*n* = 16) indicated an increase in the frequency of use of student-centered strategies, particularly use of immediate response devices (e.g., clickers), small group activities, and individual student work; as well as increases the use of pre-class assignments, reflective activities (e.g., 1-min paper, students reflecting on lecture material, think-pair-share), and whole-class discussions (Fig. 2A). For example, 73% of the 16 responding Faculty Scholars indicated they used clickers every class in their reformed course (i.e., most recent occasion) versus 29% that used them every class before the reform (i.e., oldest occasion in the last 5 years) (see Additional file 2: Fig. S1). About 68% out of 16 responding Faculty Scholars indicated that they used small group activities and individual work in every class, after the reform, compared to about 6–13% before the reform (Additional file 2: Fig. S1). CISL faculty respondents also indicated a decrease in direct instruction practices, particularly lecturing and showing slides (e.g., PowerPoint). There was a decrease in the reported frequency of lecturing from 88% of faculty respondents indicating they lectured every class before the reform to 53% after the reform, and a decrease from 69% to 38% showing slides every class (*n* = 16 Faculty Scholar respondents). In addition, 20% of the faculty respondents self-reported that they never lecture (compared to 6% before the reform). When comparing ChIPP responses from Faculty Scholars to those from a national sample of instructors at peer institutions teaching a similar range of content-area courses, Faculty Scholars self-report implementing student-centered instructional practices at a higher frequency and instructor-centered instructional practices at a lower frequency (Additional file 2: Fig. S1). Combined, these results suggest that Faculty Scholars implemented increased levels of evidence-based instructional practices after participation in the CISL program.

#### Faculty Scholars’ extent of use of evidence-based instructional practices

Classroom observation conducted using a modified COPUS protocol (Smith et al., 2013) for a subset of Faculty Scholars (*n* = 14) between 2014 and 2020 revealed that Faculty Scholars, on average, spend about 46.2% of time using instructor-centered instructional practices, and 63.7% of time using student-centered activities (Table 3). The observation analysis suggests that Faculty Scholars dedicated a significant portion of their class to student-centered instruction. Faculty Scholars implemented a range of student-centered instructional practices across their reformed courses (see Additional file 1: Table S2). Interviews of Faculty Scholars indicated similarities and differences in the level and type of active learning strategies implemented across the 30 transformed courses. All transformed courses, barring the Biology laboratory courses, utilized undergraduate Learning Assistants (LAs) to aid implementation of active learning strategies. Most transformed courses can be described as having a partially to fully inverted course structure (DeLozier & Rhodes, 2017), in which faculty utilized embedded videos of course content (e.g., lectures) on their online learning management systems and used more student-centered approaches in class (e.g., classroom response systems such as clickers, group work facilitated by LAs, live demonstrations with worksheets, etc.). An interviewed Faculty Scholar noted, *“… our team developed a set of pre-class and in-class worksheets that are used to present the content of the course. Students complete the pre-class worksheets that often review prerequisite knowledge, before coming to class. Using the pre-class content, students work in groups on in-class worksheets to cover intended material. Lecturing is reduced to a minimum.”*

#### CISL course reforms impact on student outcomes

Student outcomes of all courses transformed under the CISL program were assessed through a regression model predicting students’ numerical final course grade (i.e., GPU, grade point unit) in CISL treatment and non-CISL groups (Table 4). CISL treatment was used to predict the outcome variable, with treatment defined as a course transformed under the CISL program, taught by a Faculty Scholar, and supported by Learning Assistants. We used historical records of the same courses taught by the Faculty Scholars before the redesign as the non-CISL comparison group. The regression included control variables that performed as expected (Table 4): (1) High-school GPA is highly predictive of college success (Allensworth & Clark, 2020) and, in our study, showed a statistically significant positive effect on the outcome variables; (2) race/ethnic minority status had a statistically significant negative effect on the outcome variables, which aligns with the well-documented evidence on achievement gaps for minoritized students (Theobald et al., 2020); (3) student sex did not have a statistically significant effect in the model (*p* > 0.01) which may reflect the conflicting results often seen in studies of gender differences in academic achievement (Eddy & Brownell, 2016); and (4) transfer student status had a statistically significant negative effect on the outcome variables, aligning with the general agreement that transfer students experience greater academic challenges, due to transfer shock and other barriers (Lakin & Elliott, 2016). STEM major status had statistically significant positive effect on GPU, with STEM students showing improved academic outcomes. The CISL program had a positive, statistically significant effect on students’ overall course numerical final grade (i.e., grade point unit). Students who enrolled in a CISL program course had, on average, 0.20 grade points higher than their counterparts enrolled in the course before the CISL redesign (*p* < 0.001, small effect size, *d* = 0.2), demonstrating a positive impact on students’ course grades (Table 4). In addition, CISL-reformed courses had higher passing rates with, on average, 9.6 percentage point difference and 14.5% more students passing the course (Table 5).

**Table 4:** Regression results of CISL Program treatment on student Course Grade Point Unit

Variables	Estimate	Std. error	*p*-adj
(Intercept)	− 0.137	0.0413	***
CISL Program treatment	0.203	0.0124	***
Controls			
Student Sex (Male)	− 0.028	0.0116	*
Student racial/ethnic minority status	− 0.112	0.1518	***
Student transfer status	− 0.166	0.0128	***
Student major (STEM)	0.181	0.1193	***
Student high-school GPA	0.622	0.0094	***
Adjusted *R*-squared	0.11		
*N*	55,713 records		

*ns* not significant (*p* > 0.01), **p < 0.01, ***p < 0.001

**Table 5:** CISL Program treatment effect on course passing rate

	Average pass rate in Faculty Scholars CISL courses		Percent change in passing rate
	Before CISL redesign	After CISL redesign	
All CISL courses	66.0% (*n* = 20,705)	75.6% (*n* = 43,223)	14.5%

### Research question 3: elements of the CISL program that best supported Faculty Scholar’s course transformations and were identified to be critical to sustaining course transformations

To understand how the CISL program influenced and supported Faculty Scholars’ course transformation efforts, interviews of Faculty Scholars and administrators were conducted in 2015 and 2018. These interviews were followed by a survey in 2020 asking Faculty Scholars to rate how important the different elements of the program were in supporting their transformation efforts and sustaining the changes. Elements in the survey included both personnel support (e.g., Learning Assistants, CISL Assistant Director, Fig. 3), financial support (e.g., summer salary, Fig. 4), and community support (e.g., support from Discipline-Based Education Research faculty, Fig. 5). The emergent themes from the interviews and the survey indicate that the program Assistant Director and the Learning Assistants played the biggest roles in their transformation efforts, followed by their interaction with Discipline-Based Education Research (DBER) community at the university.

#### CISL program personnel support—Assistant Director

The CISL program Assistant Director played a key role as a communication link between evidence-based teaching practices and the Faculty Scholars (see implementation-based strategy, Fig. 1): About 75% of Faculty Scholars rated their “interaction with the Assistant Director” to be important or very important for course transformation; with a rating of 3 or more on a 0–4 scale. A majority of the respondents agreed with statements that the Assistant Director supported their needs during and after their CISL course transformation, with over 80% of respondents agreeing to such statements with the maximum rating and over 95% agreeing with a rating of 3 or more, on a 0–4 scale (Fig. 3). In addition, Faculty Scholars reported that they were comfortable reaching out to the Assistant Director during and after their transformation, with 90% of respondents giving the highest rating of agreement. Over 80% of respondents indicated that “assistance in data collection” and “assistance in data analysis”, both key roles of the Assistant Director in the implementation stage, were important or very important to their course transformation efforts (Fig. 3). Over the course of the CISL program, three Assistant Directors were hired. All three Assistant Directors were early career scholars with a Ph.D. in a STEM discipline and demonstrated experience in STEM education not exceeding 1 year.

#### CISL program personnel support—Learning Assistants

All course transformations, except the laboratory course reforms that were supported by graduate Teaching Assistants, capitalized on the undergraduate Learning Assistant (LA) program in facilitating active learning strategies during class time. All Faculty Scholars interviewed in 2015 and 2018 agreed that the most notable support provided by the CISL program was the use of LAs and stated that active learning in their classrooms would not be feasible without their extra support. Faculty Scholars noted that the LAs allow the instructors to interact with students on a personal level, which is often impossible in “traditional” large enrollment courses. In addition, administrators and Faculty Scholars pointed out that LAs balanced out the lack of representative diversity among faculty. As one administrator noted, *“The diversity of faculty doesn’t represent student diversity. That’s where the LAs have been helpful to balance.”* Over 85% of Faculty Scholars who responded to the survey in 2020, also agreed that LAs were an integral part of their ability to transform their course, with a rating of 3 or more on a 0–4 scale, while only about 6% responded that they were “not at all” important (Fig. 3). It is important to note that two laboratory course reforms did not include any LAs in their transformation and these responses may be included in the survey respondents. Moreover, 98% of Faculty Scholars gave the “financial assistance for Learning Assistants” the highest rating among the programmatic support elements provided by the CISL program in transforming their courses; with over 80% and 95% giving the rating of 3 or 4, respectively, on a 0–4 scale (Fig. 4). This result is in agreement with the responses from Faculty Scholars interviewed in 2015 and 2018 who expressed that the success of the program heavily depended on the continued funding of LAs in their classrooms. As one Faculty Scholar noted *“The only way to continue [transforming our classes] is if we have LAs. Handling the team activities even if I only have a 1/4 of my normal 500 students in one course, it’s just not possible without LAs”*.

#### CISL program financial support

Along with financial support for LAs, the CISL program also provided Faculty Scholars with other financial incentives during the design and implementation stages to aid them in their course transformation efforts. These incentives included 3 weeks of summer salary during course design, a salary overload (i.e., extra salary compensation) during two semesters of course implementation, as well as access to summer salary in the form of research supplements and conference travel support to conduct data analysis and present course transformation results. Of the different types of incentives provided, Faculty Scholars ranked the two-semester overload provided during the implementation stage the highest, with 94% of respondents ranking it important or very important (Fig. 4). Similarly, the summer salary provided during the design stage was also highly ranked, with 88% of Faculty Scholars stating it was important or very important for supporting their course transformation (Fig. 4). Travel awards to attend national conferences and/or workshops and research salary supplements to encourage publication of course transformation results were deemed to be relatively less important financial incentives with about two thirds of respondents rating it as important or very important, respectively (Fig. 4). These last two types of financial incentives were optional, with about 20% of Faculty Scholars requesting and being provided summer research supplement and about 30% requesting and being supported to attend regional and national conferences to present their course reforms. A total of eight Faculty Scholars have attempted to convert their course transformations into journal articles, with two Faculty Scholars successfully publishing their results (Gavassa et al., 2019; Rein & Brookes, 2015) in peer-reviewed journals and two additional ones currently finalizing their manuscripts for journal submission.

#### Institutional faculty community support

A key element that influenced Faculty Scholars’ course transformation efforts was the presence of a community of STEM education advocates at the university, which had been promoted by recent institutional hires of tenure-track Discipline-Based Education Research (DBER) faculty as well as a regular STEM education seminar series. These elements were initiated or promoted by the CISL program (including through funding two DBER faculty’s salary) and have now been institutionalized. All Faculty Scholars interviewed credited the seminar series for increased collaboration and communication within each department. In the survey, more than 80% of Faculty Scholars agreed that interaction with the faculty community participating in the seminar series was important for their course transformations (Fig. 5). When given the opportunity to detail how this interaction impacted their course redesigns through open response items, one common theme was that tenure-track DBER faculty provided ideas on how to implement teaching strategies and assessments of active learning in their classrooms. For example, one Faculty Scholar stated: *“My interaction with [DBER faculty member] has been tremendously impactful in my course transformation. [DBER faculty member]'s knowledge of the literature has been very helpful. [They have] shared with me articles demonstrating the effectiveness of teaching pedagogies and encouraged me to try them. [They have] also been extremely helpful in thinking of ways to assess and measure the impact of my transformations on the success of the students.”* One Department Chair also acknowledged the impact of this support saying, *“The DBER group is strong and meets weekly to discuss best practices, share resources, and present their own innovation…those are the originators of the ideas.”* Besides interactions with the DBER community, Faculty Scholars also interacted with faculty within and outside their departments. A majority of Faculty Scholars discussed evidence-based teaching practices with other faculty members within their departments, with over 80% of respondents reporting they engage in this practice (rating of 3 or 4, in 0–4 scale) (Fig. 5). Faculty Scholars also reported discussing instructional practices with faculty outside their department, with over 70% agreeing to this statement with a rating of 3 or higher (in a 0–4 scale).

#### Departmental influence

The culture of Faculty Scholar’s home departments was a factor in motivating and sustaining their course transformation. On the survey, about 60% of the respondents, overall, indicated that departmental culture played a role in motivating them to transform their courses, by giving it a rating of 3 or 4 on a 0–4 scale; compared to about 20% of respondents that stated that departmental culture did not play a significant role (0 and 1 ratings, 0–4 scale). Individual departments had a differential role on Faculty Scholars’ motivations to transform courses, with some departments having a positive impact while others a neutral or negative one. For example, a Faculty Scholar from a supportive department mentioned that *“they were very supportive and understanding of the changes. Realizing that my student evaluation might be a little more negative during the initial changes.”* In contrast, a response from a Faculty Scholar from an unsupportive department stated that *“sadly, my departmental culture is somewhat against interactive/student-centered teaching methods, so it [i.e., departmental culture] had no impact. I did it in spite of [the] department culture.”*

Most Faculty Scholars sustained their course transformations and a majority expanded student-centered instruction to other courses. One of the major objectives of the CISL program was to assist Faculty Scholars in re-designing their courses in a way that they can be sustained as well as create environmental structure that will promote future course transformations. About 75% of Faculty Scholars survey respondents indicated that they had continued the original CISL course redesign after the completion of their two-semester implementation, and 63% responded that they had expanded transformations to other courses that were not originally supported through the CISL program. Some of the main reasons cited for not being able to sustain the course reform were extrinsic to Faculty Scholar control, including no longer being assigned that particular course, or a course being cancelled or moved to online format. Some Faculty Scholars cited other reasons that informed their choice, such as structural obstacles (e.g., a transformed course needing additional class time to incorporate active learning) or student success concerns (e.g., some students not performing successfully in subsequent courses).

Through open-ended questions on the survey, the Faculty Scholars were asked to reflect on what they deemed as necessary support from their respective departments and from the university to be able to sustain course transformations. Emergent themes included instating institutional structures required for supporting and rewarding faculty instructional changes. Support structures that were mentioned included financial support in the form of salary overload during course reforms and support for hiring LAs to facilitate student learning and faculty change. These results were also supported by responses from interviews of Department Chairs and Faculty Scholars conducted in 2015 and 2018, where both groups acknowledged that the financial support and course flexibility were vital to successfully sustaining course reforms. Interviews of administrators indicated that across the institution, both Department Chairs and the institution’s administration understood that providing flexibility to Faculty Scholars was necessary to properly develop the transformed courses and have adequate time and space for implementation, assessment, and corrections. As one Department Chair acknowledged, *“I need to be as flexible as possible with scheduling to allow them to be able to try these new techniques. These things take time to prepare, implement, and then to assess afterwards. That way they can find out what works and what doesn’t.”* Institutional reward mechanisms included reframing the evaluation of teaching in promotion and tenure process to rewarding excellent teaching and encouraging faculty to undertake new and sustain original course transformations. Faculty Scholars also provided some specific suggestions of departmental support such as providing mentorship opportunities from faculty that are experienced in using evidence-based teaching techniques and increasing exposure of faculty to evidence-based teaching and its impact on student performance.

## Discussion

The CISL program provides an example of significant effort sustained over several years to systematically improve the quality and culture of undergraduate education in a large research-intensive Hispanic Serving Institution. In this study, we provide evidence of faculty change towards increased use of evidence-based instructional practices and its positive effect on student performance as an impact of the CISL program. Moreover, we identify key elements that were fundamental to providing maximum support to faculty participating in the program. The main objective of the CISL program was to promote change in faculty instructional practices through increased adoption of active-learning strategies, and our results suggest that institutional level support structures may be essential in bringing about this change. Studies have shown that there are multiple barriers that prevent faculty from changing their mode of instruction, such as insufficient training in evidence-based teaching practices (Ebert-May et al., 2011; Handelsman et al., 2004; Hativa, 1995; Miller et al., 2000; Rushin et al., 1997; Yarnall et al., 2007), lack of time for course reform (Shadle et al., 2017), and lack of incentives to reform teaching practices (Brownell & Tanner, 2012; Gess-Newsome et al., 2003). Faculty Scholars supported through this program almost unanimously agreed that support structures such as Learning Assistants (LAs), financial incentives (e.g., summer salaries for course redesign and LA hiring), and interaction with the program Assistant Director and Discipline-based Education Research (DBER) community were key to changing and sustaining innovative instructional practices. Below we discuss some of the implications of our findings and make recommendations for changes at the institution-level that can foster adoption of evidence-based teaching practices at a larger scale.

Our analysis of faculty motivation to redesign courses revealed that Faculty Scholars generally demonstrated student-centered and faculty-centered motivations. Self Determination Theory categorizes such motivations as intrinsic motivators, where internal drivers, such as core values, interests, and personal sense of morality inspire faculty to change their instructional practices (Deci & Ryan, 1985). Studies have shown that intrinsic motivations, such as developing stronger students; increasing student learning; deriving satisfaction from teaching are strong predictors of faculty change especially change in their instructional practices (Johnson et al., 2015; Orr et al., 2009; Shadle et al., 2017). We also identified some level of faculty dissatisfaction with current teaching practices which has been shown to be critical in driving pedagogical changes; as without such dissatisfaction there is very little impetus to change current practices (Gess-Newsome et al., 2003). While research shows that decision to change one’s teaching occurs at the individual level (Andrews & Lemons, 2015; Dormant & Lee, 2011; Gess-Newsome et al., 2003), faculty motivations can be leveraged proactively by departments and institutions to catalyze change. For individual faculty to be successful in changing their instructional practices, it is important to evaluate the context of the larger departmental system, as some barriers and drivers of faculty change have been reported to be department specific (Shadle et al., 2017). In addition to differences in barriers and drivers, there may be differences in faculty awareness and the extent of adoption of student-centered practices between STEM departments (Lund & Stains, 2015). Thus, department-specific actions can be planned by first assessing the instructional climate within a department, the factors motivating the faculty and the extent of faculty use of evidence-based teaching practices (Landrum et al., 2017; Sturtevant & Wheeler, 2019).

Departmental culture can be a key element that influences motivation to transform, and also sustain, course transformations (Owens et al., 2018; Reinholz et al., 2019; Shadle et al., 2017). Our findings suggest that departments can enhance faculty motivations by offering opportunities for faculty to engage in pedagogy related professional development activities; providing assistance in collecting and analyzing institutional data to inform teaching practices; or offering incentives for faculty to collaborate and co-teach courses with colleagues. The success of the CISL program, to an extent, may be attributed to the fact that its elements work towards supporting some of the motivations faculty indicated as important. For example, a number of Faculty Scholars displayed an interest in improving student metrics in their courses and the CISL program provided them with assistance in collecting and analyzing these student data. Such support can be offered by institutions and departments through hiring or developing research personnel that provide assistance to faculty in collecting data and/or utilizing institutional data resources to inform their teaching. It is crucial for departments and institutions to note that faculty’s perceived supports are more strongly related to their adoption and implementation of evidence-based teaching practices than their perceived barriers (Bathgate et al., 2019). These perceived supports include the influence of department culture, such as faculty’s perceptions of the level of support for evidence-based teaching by their departments and colleagues (Bathgate et al., 2019). Faculty resistance may be mitigated by supporting faculty members that are already inclined to implement evidence-based strategies, which may signal increased departmental receptivity to hesitant faculty and progressively lead to additional faculty adoption. For example, in the CISL program, Faculty Scholars may have inadvertently influenced non-Faculty Scholars in their departments to consider evidence-based teaching practices and submit their own application to the programs, as suggested by several Faculty Scholars commenting on their applications that interacting with active learning faculty adopters had a positive influence.

A central finding to emerge from our study was Faculty Scholar’s perceived importance of Learning Assistants (LAs) in the transformation of their courses. Although active learning has been shown to increase student performance, it can be challenging to implement fully in STEM courses, particularly those that are high-enrollment or have high student to instructor ratios. Implementation of an undergraduate Learning Assistant program is one strategy to increase engagement with students as it has been found to be effective in promoting student learning, engagement and satisfaction, as well as improving student retention (Groccia & Miller, 1996; Jardine et al., 2020; Knight et al., 2015; Pivkina, 2016; Talbot et al., 2015). In addition, studies have shown that incorporating LAs not only improves student performance but also supports course redesign efforts by faculty (Jardine et al., 2020; McHenry et al., 2010; Pavlacic & Buchanan, 2017). LAs, who recently have taken the course, provide faculty with feedback from a student’s perspective on their course redesign, which has been shown to increase faculty satisfaction with their courses. Additional benefits to involving students in course re-design efforts include increasing knowledge about course design strategies and pedagogical teaching methodologies of both the faculty and students (Healey et al., 2016; McHenry et al., 2010; Werder & Otis, 2010). Student perspectives can be incorporated through several mechanisms depending on institutional context. Institutions can take advantage of the various ways in which the Learning Assistant model can be adopted for their own use depending upon their scale of adoption and budget, as indicated by the well-established Learning Assistance Alliance (n.d.). Outside of the LA model, departments can also encourage faculty members to involve students in course transformations through the use of student consultants or by incorporating student feedback intentionally in their courses (Bunnell & Bernstein, 2014). Some institutions incorporate student consultants who carry out regular classroom observations and provide detailed feedback to faculty (Cook-Sather & Motz-Storey, 2016). Such student-faculty partnerships have been shown to be highly effective, as student consultants can often offer insights regarding readings and assignments based on previously having completed a course and explore what is happening and what could happen in faculty members’ classrooms to maximize student engagement and learning (Cook-Sather, 2014; Crawford, 2012; Cross, 2014; Mihans et al., 2008).

Another important element of the CISL program was the support Faculty Scholars received from the program’s Assistant Director. The Assistant Director’s main role was to act as a specialist that bridges the gap between Faculty Scholars and the resources on evidence-based instructional practices and supports Faculty Scholars in adopting these practices. One of the commonly cited barriers for faculty adoption of innovative instructional practices is insufficient faculty training and, thus, consulting with specialists has shown to benefit faculty in their efforts to transform their courses (Chasteen et al., 2015; Piccinin, 1999; Wieman et al., 2010). Duties of the Assistant Director included helping Faculty Scholars identify areas for improvement, providing consultation on best practices, providing regular feedback on course-specific activities or curricula, conducting classroom observations, assisting Faculty Scholars in data collection and analyses, and reporting classroom data to Faculty Scholars. These roles are well suited for a STEM education specialist but may also be fulfilled by non-STEM faculty developers and education specialists situated at different units of the institution (e.g., Centers for Teaching and Learning). In addition, the CISL program demonstrates that early career scientists with interest and minimal experience in STEM education can effectively support faculty reform efforts, as the CISL Assistant Directors were all recent STEM doctoral graduates with no more than a year of experience in STEM education. Faculty developers at Centers of Teaching and Learning (CTLs) may play critical roles by supporting faculty members in establishing student learning goals and helping integrate assessment into their teaching practice, as well as using the results to improve student learning (Kinzie et al., 2019). Moreover, these units are in a distinctive position to create communities of practice for faculty members or Faculty Learning Communities that work together on redesigning courses and identifying relevant strategies. These faculty communities allow repeated practice and reflection and provide the opportunity to discuss and implement change as a part of a group, rather than in a vacuum (Ebert-May et al., 2011; Henderson et al., 2011; Tierney, 2010). Other models of forming communities of practice, which can be adopted at the department level, involve collaborative teaching and paired-teaching programs, where instructors teach one semester in pairs and share course redesign goals and feedback (Auerbach & Schussler, 2017; Holland et al., 2018; Pelletreau et al., 2018).

CTLs can also be complemented with STEM education centers (SEC) which work towards improving the quality of teaching and learning in STEM education, broadening participation and opportunities in STEM for all students, and the expansion of institutional infrastructure and policies to support STEM learning experiences (Carlisle & Weaver, 2020). Collaborations between CTLs and SECs and other departments within the institution can lead to institutional level initiatives that support faculty in identifying trends of classroom struggles across departments and disciplines and meeting them with proposed solutions (Levesque-Bristol et al., 2019). Often, CTLs face the challenge of being overworked and under-staffed. To mitigate this, undergraduate students or graduate students can be trained in classroom observations and data collection and analyses and can be recruited to provide support to faculty (Cook-Sather, 2014; Handelsman et al., 2004; Rushin et al., 1997). Our data also highlighted that Faculty Scholars benefitted from their interaction with Discipline-Based Education Researchers (DBER) at the institution. It has been shown previously that increasing institutions’ research capacity in STEM education by hiring or developing DBER or science faculty with education specialties (SFES) can enhance faculty communities, as experts can act as facilitators of discussions and promote teaching as a scholarly endeavor (Pelletreau et al., 2018).

Not surprisingly, our study found that faculty value compensation for their efforts in the form of lower teaching loads and financial benefits, such as course overloads and summer salaries. They also feel there is a need to recognize their efforts through teaching awards and reframing the evaluation of teaching in the promotion and tenure process in order for them to sustain the changes. Faculty have many demands on their time which makes it harder for them to find the motivation and/or the time required for changing their instructional practices. It is critical for departments and institutions to recognize this obstacle; and provide corresponding flexibility in schedules (Auerbach & Schussler, 2017; Brownell & Tanner, 2012). We propose that departments can offer course reduction or small grants to faculty to explore and implement evidence-based teaching practices when the perceived barrier is lack of time. In addition, institutions should provide incentives to faculty for their effort and time, and work towards creating an environment where research and teaching is equally valued and well-integrated (Brownell & Tanner, 2012; Henderson et al., 2010; Porter et al., 2006).

## Conclusions

Overall, this work points to the importance of designing holistic faculty development programs to promote adoption of evidence-based student-centered teaching practices. Supporting faculty with programming that aligns with their motivations may lead to changes in faculty behavior and, therefore, departmental and institutional level changes. Our findings suggest that faculty valued individualized support in the form of one-on-one interaction with the program’s Associate Director and undergraduate Learning Assistants; as they provided outside perspective, constructive feedback, and access to resources. Financial incentives to protect faculty time, compensate additional work, and lower teaching loads (e.g., summer salary support) were also valued by faculty. Departmental and institutional culture also plays a critical role in supporting faculty change and, thus, creating opportunities to enhance drivers and reduce obstacles at these levels can have a critical impact in promoting adoption of student-centered instructional practices.

## Supplementary Information

**Additional file 1: Table S1.** Faculty Scholars responded to items in the ChIPP survey (Benabentos et al., 2020), as shown in Fig. 2. Faculty Scholars rated how important specific elements of the HHMI Faculty Scholar Program were to supporting their course transformation, as shown in Figs. 3, 4 and 5. Faculty responses were on a 5-level anchored scale with rating 4 indicating highest importance/agreement (i.e., “very important”) and rating 0 indicating lowest importance/agreement (“not at all important”). Faculty Scholars were observed using COPUS protocol (Smith et al., 2013) and instructor and student-centered behaviors were coded, as shown in Table 3. **Table S2.** Summary of reformed courses through CISL program.

**Additional file 2: Figure S1.** Frequency of use of instructional practices by Faculty Scholars and a STEM faculty national sample. This figure shows frequency of use of particular instructional practices for a sample of Faculty Scholars (either before or after redesign) and a national STEM faculty sample at comparable institutions. Faculty reported the use of these practices for the STEM course they most frequently teach. Each row represents a particular instructional practice surveyed and each column represents the reported frequency of use of that particular practice. The calculated percentages for each individual instructional practice do not include faculty who did not provide a response for that survey item (i.e., NA responses). The top four instructional practices (marked with an asterisk) on the *y*-axis were considered to be instructor-centered (i.e., not student-centered). Each row is independent and accounts for all responses to that particular survey item. For example, 53% of surveyed Faculty Scholars (after reform) reported that they lecture every class, while 20% of them reported that they never lecture.

**Additional file 3.** HHMI CISL Program. Semi-structured Administrator/Faculty Interview Protocol.

## Data Availability

The data that support the findings of this study are available upon reasonable request from the corresponding author, RB and following IRB restrictions. The data are not publicly available due to their containing information that could compromise the privacy of research participants.
