# Supplementary material for: Institutionalizing evidence-based STEM reform through faculty professional development and support structures
Source: Int J STEM Educ. 2022 May 12;9(1):36. doi: 10.1186/s40594-022-00353-z (PMC9098573; doi:10.1186/s40594-022-00353-z)
Supplement: Supplementary file 1 — Additional file 1: Table S1. Faculty Scholars responded to items in the ChIPP survey (Benabentos et al., 2020), as shown in Fig. 2. Faculty Scholars rated how important specific elements of the HHMI Faculty Scholar Program were to supporting their course transformation, as shown in Figs. 3, 4 and 5. Faculty responses were on a 5-level anchored scale with rating 4 indicating highest importance/agreement (i.e., “very important”) and rating 0 indicating lowest importance/agreement (“not at all important”). Faculty Scholars were observed using COPUS protocol (Smith et al., 2013) and instructor and student-centered behaviors were coded, as shown in Table 3. Table S2. Summary of reformed courses through CISL program. [file 40594_2022_353_MOESM1_ESM.pdf]

Supplementary Table 1

Figure	Figure axis label	Full statement	Reference
2	Clickers	I collected responses from students in real time in class (e.g. flashcard, clicker, raising hand)	Benabentos et al. 2020
2	Individual work	Students did individual work in class	Benabentos et al. 2020
2	Pre-class assignments	I gave students assignments or quizzes on the readings/videos prior to covering material in class	Benabentos et al. 2020
2	Reflective activities	I asked students to engage in reflective activities (e.g. one minute paper, students reflecting on lecture material, think-pair-share)	Benabentos et al. 2020
2	Small group activities	Students did small group activities	Benabentos et al. 2020
2	Whole-class discussion	Whole-class discussion	Benabentos et al. 2020
2	Lecture*	Lecture	Benabentos et al. 2020
2	Show slides*	I showed slides (e.g. PowerPoint)	Benabentos et al. 2020
2	Videos, demos, and simulations*	I showed videos, simulations, or demonstrations related to the material	Benabentos et al. 2020
2	Wrote on board*	I wrote/solved problems on a chalk or white board	Benabentos et al. 2020
3	Learning Assistants support during reform	The Learning Assistants were an integral part of my ability to transform my course under HHMI.	
3	Assistant Director support during reform	The HHMI Assistant Director supported my needs **during** my HHMI course transformation.	
3	Assistant Director support after reform	The HHMI Assistant Director supported my needs **after** my HHMI course transformation.	
3	Support for data collection	Assistance in data collection	
3	Support for data analysis	Assistance in data analysis	
3	Assistant Director interactions	Interaction with Assistant Director	
4	Conference travel support	Travel awards	
4	Summer research supplement	Summer research supplement after implementation	
4	Summer salary during design	Summer salary	
4	Overload salary during implementation	Overload during implementation semester	
4	LA financial assistance	Financial assistance for Learning Assistants	
5	Motivation from departmental culture	Departmental culture played a role in motivating me to transform my course under HHMI.	
5	Evidence-based teaching discussions within department	I discuss evidence-based instructional practices with faculty **within** my department.	
5	Evidence-based teaching discussions outside department	I discuss evidence-based instructional practices with faculty **outside** my department.	
5	DBER community interactions	Interaction with DBER community e.g. DBER seminar series was important in supporting my course transformation	
Table 3	Instructor-centered Instructional Practices	Lecture	Adapted from Smith et al, 2013
Table 3	Instructor-centered Instructional Practices	Real-time writing on the board, document projector, etc.	Adapted from Smith et al, 2013
Table 3	Instructor-centered Instructional Practices	Showing and conducting a demo, experiment, simulation, video or animation	Adapted from Smith et al, 2013
Table 3	Student-centered Instructional Practices	Asking clicker questions	Adapted from Smith et al, 2013
Table 3	Student-centered Instructional Practices	Follow-up discussion after clicker question	Adapted from Smith et al, 2013
Table 3	Student-centered Instructional Practices	Posing non-clicker questions to all students (non-rhetorical)	Adapted from Smith et al, 2013
Table 3	Student-centered Instructional Practices	Listening to and answering student questions with the entire class listening	Adapted from Smith et al, 2013
Table 3	Student-centered Instructional Practices	Moving through class and looking at student work	Adapted from Smith et al, 2013
Table 3	Student-centered Instructional Practices	One-on-one discussions with an individual student	Adapted from Smith et al, 2013
Table 3	Student-centered Instructional Practices	Talking/interacting with a group of students	Adapted from Smith et al, 2013

Supplementary Table 2: Summary of reformed courses through CISL program

FS	Course Name	Course Level		Avg Section enrollment			Course type				New instructional strategies incorporated in the reformed course						
		Lower-division	Upper-division	<80	80-199	200+	Required for STEM major	Elective for major	Service course	Fulfills General Education Credit	Undergraduate Learning Assistants in class	Flipped Classroom (Partial to Fully)	In-class problem-based learning	In-class small group work	Additional formative assessments (individual)	Clicker questions	Other new strategies incorporated
**Biology**																	
FS1	General Biology I	✓				✓	✓			✓	✓		✓	✓	✓		Hybrid course (Gavassa et al, 2018).
FS2	General Biology II	✓				✓	✓			✓	✓		✓	✓		✓	
FS3	Genetics		✓			✓	✓				✓	✓	✓	✓			Project-based learning, student presentations
FS4	Genetics		✓			✓	✓				✓	✓	✓	✓	✓	✓	Case studies
FS5	Genetics		✓			✓	✓				✓	✓	✓	✓	✓	✓	Case studies, in-class worksheets for students to complete in small groups
FS6	Ecology		✓	✓			✓				✓	✓	✓	✓			
FS7	Evolution		✓			✓	✓				✓	✓		✓	✓		Writing for learning projects
FS8	Evolution		✓			✓	✓				✓	✓		✓			
FS9	General Biochemistry		✓			✓		✓			✓	✓	✓	✓	✓	✓	
FS10	Coral Reef Ecology		✓	✓				✓			✓	✓	✓	✓			Project-based learning, student presentations
FS11	Human Anatomy		✓			✓		✓			✓	✓	✓	✓	✓	✓	
FS12	Bioinformatics		✓	✓				✓			✓		✓	✓			Project-based learning, course-based research project, writing for learning, student presentations
FS13	Epigenetics		✓	✓				✓			✓	✓		✓		✓	Small group discussions of primary literature, in-class worksheets for students to complete in small groups
FS14	Genetics Laboratory		✓	✓				✓					✓	✓			Course-based research project
FS15	Evolution Laboratory		✓	✓				✓					✓	✓			Course-based research project
**Chemistry**																	
FS16	Survey of Chemistry	✓			✓				✓	✓	✓						
FS17	Survey of Organic Chemistry	✓			✓				✓		✓		✓	✓		✓	Rein and Brookes, 2015
FS18	General Chemistry I	✓				✓	✓				✓		✓	✓			
FS19	General Chemistry I	✓				✓	✓				✓	✓	✓	✓	✓	✓	POGIL-like worksheets for students to complete in small groups.
FS20	General Chemistry II	✓				✓	✓				✓	✓	✓	✓	✓	✓	Partially flipped classroom: incorporated LAs and in-class POGIL-like worksheets for students to complete in small groups.

FS21	Organic Chemistry I	✓				✓	✓				✓	✓	✓	✓			Partially flipped classroom: incorporated LAs and in-class worksheets for students to complete in small groups.
FS22	Biological Chemistry		✓		✓			✓			✓	✓	✓	✓		✓	Partially flipped classroom: incorporated LAs and in-class worksheets for students to complete in small groups.
FS23	Inorganic Chemistry		✓	✓				✓			✓		✓	✓			Pre-class worksheets with online resources
FS24	Advanced Inorganic Chemistry		✓	✓				✓			✓		✓	✓			Pre-class worksheets with online resources
**Mathematics**																	
FS24	Intermediate Algebra	✓			✓				✓		✓		✓	✓	✓		Pre-class, in-class and homework worksheets aligned. Feedback provided. Supplemental instruction offered.
FS25*	Pre-Calculus Algebra	✓		✓						✓	✓		✓	✓	✓		
FS26*	Calculus I	✓		✓			✓			✓	✓		✓	✓	✓		
**Physics**																	
FS27	Stellar Astronomy	✓			✓				✓	✓	✓		✓	✓			
FS28	Physics without Calculus	✓			✓		✓				✓		✓	✓	✓	✓	Competency-based learning
FS29	Physics with Calculus	✓			✓		✓				✓		✓	✓			
FS30	Modern Physics		✓	✓			✓				✓		✓	✓			
