# Supplementary figures and images for: Institutionalizing evidence-based STEM reform through faculty professional development and support structures
Source: Int J STEM Educ. 2022 May 12;9(1):36. doi: 10.1186/s40594-022-00353-z (PMC9098573; doi:10.1186/s40594-022-00353-z)

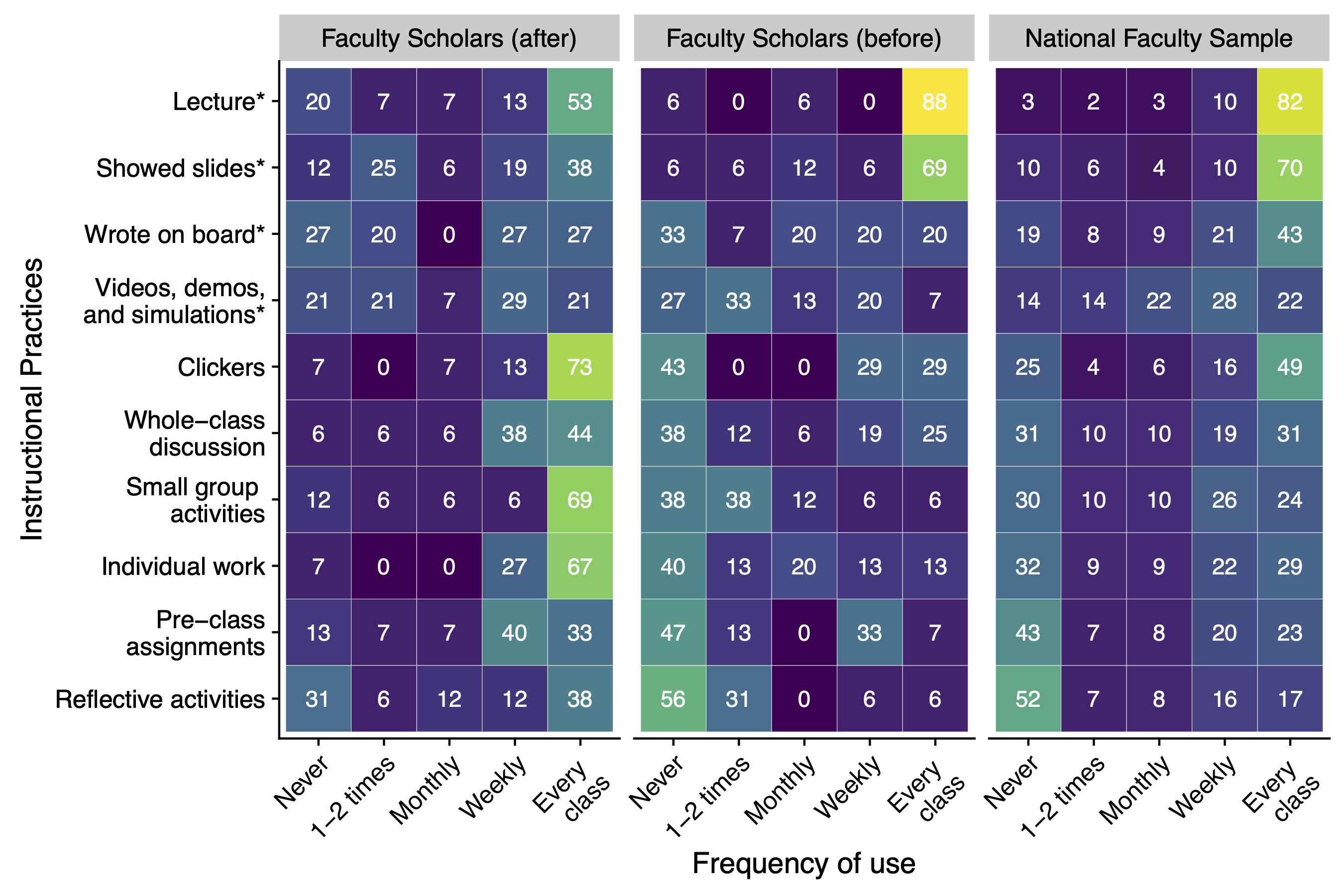

Supplement: Supplementary file 2 — Additional file 2: Figure S1. Frequency of use of instructional practices by Faculty Scholars and a STEM faculty national sample. This figure shows frequency of use of particular instructional practices for a sample of Faculty Scholars (either before or after redesign) and a national STEM faculty sample at comparable institutions. Faculty reported the use of these practices for the STEM course they most frequently teach. Each row represents a particular instructional practice surveyed and each column represents the reported frequency of use of that particular practice. The calculated percentages for each individual instructional practice do not include faculty who did not provide a response for that survey item (i.e., NA responses). The top four instructional practices (marked with an asterisk) on the y-axis were considered to be instructor-centered (i.e., not student-centered). Each row is independent and accounts for all responses to that particular survey item. For example, 53% of surveyed Faculty Scholars (after reform) reported that they lecture every class, while 20% of them reported that they never lecture. [file 40594_2022_353_MOESM2_ESM.tiff]
