# Supplementary material for: Institutionalizing evidence-based STEM reform through faculty professional development and support structures
Source: Int J STEM Educ. 2022 May 12;9(1):36. doi: 10.1186/s40594-022-00353-z (PMC9098573; doi:10.1186/s40594-022-00353-z)
Supplement: Supplementary file 3 — Additional file 3. HHMI CISL Program. Semi-structured Administrator/Faculty Interview Protocol. [file 40594_2022_353_MOESM3_ESM.pdf]

HHMI CISL Program
Semi-structured Administrator/Faculty Interview Protocol

1) What is your current role/position at the institution?
2) How long have you been at the institution?
3) Please describe in your own words your roles in the HHMI CISL project.
4) What led you to participate in the project?
5) Please describe in your words the goals of the HHMI project.
   a) To what extent were they met?
   b) How are those goals aligned with broader University policy and goals?
6) To what extent do you feel the project has developed a shared vision of the goals?
   a) How are the project's vision and goals shared with the broader University community?
7) What steps have been taken to implement the HHMI project?
   a) Would you say that the project has been implemented according to its plan and schedule?
   b) What factors aided or hindered implementation of the grant?
8) What policy changes occurred at FIU in response to HHMI1?
   a) Are there other policy changes and or infrastructural changes that need to happen to improve the likelihood of HHMI achieving its goals?
9) [Administrators] Would you say the project has been successful recruiting faculty into the project?
   a) Are you getting the participation you anticipated (numbers, tenure, racial/ethnic background)?
   b) What factors would you say have facilitated recruitment? What factors have been barriers?
   c) How has recruitment evolved?
10) What steps have been taken to sustain the HHMI project beyond the grant?
   a) Have the results from HHMI1 been disseminated internally within FIU? If so, has it generated interest from other departments or faculty?
   b) What are the plans to disseminate the HHMI work within FIU in the upcoming year?
11) How has the HHMI project changed instruction at FIU (administrators)/in your instruction (faculty)?
12) [Faculty] To what extent do you interact with other DBER faculty about
   a) Sharing instructional practices
   b) Observing your classroom
   c) You conducting observations of others' classrooms
   d) Learning about instructional practices through seminars, talks, etc
13) [Faculty] To what extent do you interact with non-DBER faculty about
   a) Sharing instructional practices
   b) Observing your classroom
   c) You conducting observations of others' classrooms
   d) Learning about instructional practices through seminars, talks, etc
14) [Administrators] Are you aware of faculty working together to share instructional practices or conducting classroom observations?
   a) Have you taken any steps to encourage that collaboration?
